# Large-scale genomic deletions mediated by CRISPR/Cas9 system

**DOI:** 10.18632/oncotarget.14543

**Published:** 2017-01-06

**Authors:** Yuning Song, Liangxue Lai, Zhanjun Li

**Affiliations:** ^1^ Jilin Provincial Key Laboratory of Animal Embryo Engineering, Jilin University, Changchun, China

**Keywords:** large-scale genomic deletions, CRISPR/Cas9 system, multiple sgRNAs, NHEJ, inhibitors

Owing to its simplicity of manipulation and high efficiency, the CRISPR/Cas9 technology has already been achieved as a robust genome engineering tool for generating gene mutations, multiple-gene knockout and knock-in in various species. However, tissues and genomic mosaicism have been widely reported in the gentically modified animals generated from zygotes that were co-injected with Cas9 mRNA and single-guide RNA (sgRNA) [1]. Further breeding was therefore necessary to obtain the desired genotype and phenotype. Furthermore, the low efficiency of conventional gene targeting strategies and a lack of mutants have hindered the functional study of gene clusters, long non-coding RNAs (lncRNA) and regulatory sequences. Alternatively, dual sgRNA-directed large gene deletion mediated by CRISPR/Cas9 system is a robust tool for completely eliminate the protein, no mosaic mutations and large deletions of lncRNA genes [2, 3].

Several recent studies have reported the CRISPR/ Cas9 system-induced large genomic deletion, ranged from 23kb to 1Mb, in mouse [4], *C. elegans* [3], rabbit [5] and human cell lines [6]. However, the major observed is that the larger the size of the deleted fragment, the lower the efficiency. In our study, although we achieved high efficiency (~80%) of deletion with each sgRNA, the efficiency of large fragment deletions was less than 10%. Therefore, improving the efficiency of large fragment deletion is a matter of cardinal significance [5].

Although paired sgRNAs can precisely generate large deletions, the deletion efficiency was doubled (from 16% to 33.3% ) when multiple sgRNAs were used [2]. Previous studies have also reported that combined use of multiple sgRNAs to target an individual gene could improve the efficiency of large fragment deletions [4]. In our study, the efficiency of a 105kb fragment deletion was increased to 17% with four sgRNAs, indicating that multiple sgRNAs can improve the efficiency of large fragment deletion in rabbit [5]. However, higher efficiency of large fragment genomic deletion is not necessarily achieved when more sgRNAs are used. In contrast, increased number of transfected or injected sgRNAs may in fact reduce the efficiency of individual sgRNAs. Therefore, we speculate that no more than four sgRNA are required for the high efficiency of CRISPR/Cas9 directed large fragment deletion.

It is well-known that the mechanism of CRISPR/ Cas9 mediated gene mutation is DNA double-strand breaks (DSB), then followed by induced Homologous Recombination (HR) and Non-Homologous End-Joining (NHEJ). NHEJ events are more predominant than HR and also correlate with the observed frequency of genomic deletions or inversions in eukaryotic genomes [7]. Thus, we hypothesize that the reason for the low efficiency of CRISPR/Cas9 directed large fragment deletions is due to the desired DSBs being repaired during NHEJ events. Reasonable inhibition of NHEJ can increase the co-action time of multiple sgRNAs. Previously study have shown that DNA-dependent protein kinase catalytic subunit (DNA-PKcs), Ku70/80, Ligase IV and XRCC4 play a key roles on NHEJ [7]. Specifics inhibitors have been designed to target differentactors of the DSB repaire pathway. Therefore, it is necessary to investigat the possibility of using small molecule inhibitors (NU7441 and KU- 0060648) that target DNA-PKcs to restrain the rates of NHEJ repair events, and as to improve the efficiency of large-scale genomic deletions by the CRISPR/Cas9 system.

Taken together, we believe that the mechanism of large-scale genomic deletion mediated by CRISPR/ Cas9 system warrants further exploration. Furthermore, the CRISPR/Cas9 system in combination with multiple sgRNAs provides a powerful platform for easy generation of large-scale deletions in the genome of an organism.
